# Prader-Willi syndrome - type 1 deletion, a consequence of an unbalanced translocation of chromosomes 13 and 15, easily to be mixed up with a Robertsonian translocation

**DOI:** 10.1186/s13039-015-0163-2

**Published:** 2015-07-22

**Authors:** Frenny Sheth, Thomas Liehr, Krati Shah, Jayesh Sheth

**Affiliations:** FRIGE’s Institute of Human Genetics, FRIGE House, Jodhpur Gam Road, Satellite, Ahmedabad, 380015 India; Jena University Hospital, Institute of Human Genetics, Kollegiengasse 10, D-07743 Jena, Germany

**Keywords:** Structural rearrangement, sSMC, Deletion, Prader-Willi syndrome, Unbalanced translocation, FISH

## Abstract

**Background:**

Prader-Willi syndrome, due to microdeletion of proximal 15q, is a well-known cause of syndromic obesity.

**Case characteristics:**

A couple with history of repeated first trimester abortions had a son with balanced Robertsonian translocation of chromosomes 13 and 15 according to cytogenetic banding technique.

**Results:**

Chromosomal analysis for the couple was performed. A balanced translocation involving BP1-BP3 region of proximal 15q was observed in the father.

**Discussion:**

Investigations of the parents is mandatory when a structural rearrangement is detected in a dysmorphic child.

## Background

Prader-Willi syndrome (PWS) is a neurobehavioral genetic disorder (OMIM #176270) characterized by hypotonia, poor feeding in infancy, hyperphagia with evolving obesity in later live, hypogonadism, decreased adult height as well as cognitive and behavioural disabilities [1]. PWS can be due to distinct genetic mechanisms: deletion of paternally expressed functional genes, maternal uniparental disomy and imprinting defects of genes in proximal 15q. Micro-deletions in PWS are further subdivided into type-1 (DT1) and type 2 (DT2). Both of them are usually due to a “*de novo”* event. Type 1 encompasses breakpoints (BP) BP1 to BP3 (~6 Mb) whereas type 2 covers BP2 toBP3 (~5.6 Mb) [2]. However, there are other rare PWS cases where 15q11.2-13 region may be deleted as a result of unbalanced translocation leading to discrepant breakpoints in proximal 15q. Various diagnostic modalities like testing of DNA methylation test or microsatellite analysis, fluorescence *in situ* hybridization (FISH) or chromosome microarray (CMA) techniques are prerequisite to undoubtedly confirm the clinical diagnosis of PWS.

Here, we present a rare case of PWS arisingas a consequence of paternally inherited unbalanced translocation involving chromosome 13 and 15 resulting in loss of proximal 15q, which can easily be misinterpreted as Robertsonian translocation.

## Case presentation

A non-consanguineous elderly couple was referredfor cytogenetic evaluation with repeated first trimester pregnancy losses (*n* = 3). In addition, there was a history ofa male child with uncontrolled seizures who died at nine months of age. Another male child, the proband of this study, expired at the age of fifteen years due to obesity leading to sleep apnea. This child had intellectual disability and hyperphagia with central obesity. He had all the typical features of PWS along with extreme impairment of language milestones and could only speak few words even at 12 years of age. On cytogenetic evaluation, he had a karyotype of 45,XY, rob (13;15)(q10;q10)[100 %]. There was no history of repeated miscarriages in any other family members; however, intellectual disability was observed in one of the members on the paternal side.

**Fig. 1:**
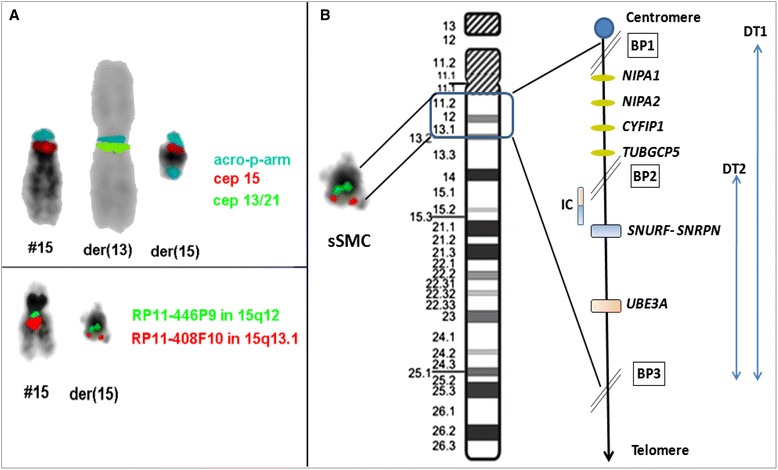
**a** Partial karyotype showing comprehensive characterization of the balanced rearrangement involving chromosome 13 and 15 in the father of the proband using various FISH probes as indicated in the figure. The paternal karyotype was thus redefined as 46,XY,t(13;15)(p11.2;q13.2). **b** Schematic diagram showing deleted region (DT1) harbouring various genes that were absent in the proband. Abbreviations: BP-Break point, IC-Imprinting Center, DT1-deletion type-1, DT2-deletion type-2

Cytogenetic analysis was carried out from the peripheral blood of the coupleto rule out inheritance of Robertsonian translocation [rob(13;15)] detected in the proband. Chromosomes were identified and classified according to the guidelines by the International System for human Cytogenetic Nomenclature (ISCN, 2013) [3]. The identical Robertsonian translocation was detected in the father along with a tiny unidentified chromosomal segment, known as small supernumerary marker chromosome (sSMC). The paternal karyotype was thus 46,XY,rob(13;15)(q10;q10),+mar[100 %], while the karyotype of the mother was normal. Further characterization of sSMC was carried out using various probes in 2 to three colour FISH settings probes for the centromeric regions of chromosomes 13/21 (D13/21Z1) and 15 (D15Z4 – both ZytoVision, Bremerhaven, Germany) together with a homemade probe for all acrocentric short arms (midi54 = acro-p-arm) and BAC probes RP11-446P9 (in 15p12) together with RP11-408 F10 in 15 q13.1. This substantiated that the sSMC was a “by-product” of a balanced paternal translocation involving one chromosome 13 and 15 and mimicking a Robertsonian translocation.Breakpoints were located in 13p11.2 and 15q13.2 region and the karyotype was redefined as 46,XY,t(13;15)(p11.2;q13.2) (Fig. 1a). Hence, the proband had an unbalanced karyotype 45,XY,der(13)t(13;15)(p11.2;q13.2),-der(15)t(13;15)(p11.2;q13.2).

## Discussion

PWS, a contiguous gene disorder results in to functional inactivation of paternally derived genes at 15q11.2-q13 region; this kind of alteration is detected in ~70 % cases. The proximal region of the long arm of chromosome 15 (15q) is rich in duplicons and thus vulnerable to genomic instability [4]. This region further houses six genomic breakpoint (BP) regions, assigned as BP1 to BP6, from the centromeric to telomeric region [5]. Each break point is surrounded by a complex set of low-copy repeats which in turn lead to a variety of genomic imbalances and subsequent rearrangements. A unbalanced translocation involving chromosomes 13 and 15 was inherited from the healthy father in the present case, as the der (15) spanning 15pter to q13.2 harbouring BP1 to BP3 region (DT1) lacked in the proband (Fig. 1b). There exists a controversy between severity of the phenotype of DT1 and DT2 deletions. Approximately, the ratio prevailing between them is 2:3 [6]. Cases with the larger DT1 (~6 Mb) have an estimated difference of 500 kb of genetic material than cases with the smaller type 2 deletion (~5.5 Mb). The BP1-BP2 region of 500 kb harbour four genes: *NIPA1, NIPA2, CYFIP* and *TUBGCP5*; those are highly conserved and implicated in developmental delay and psychological consequences since they are expressed in the central nervous system [7]. Butler et al. (2004) found that people with DT1 had more psychological and neurological deficit than people with DT2 [8]. However, Varela et al. [9] studied 75 individuals and optedthat there was no statistically significant difference between both types of deletions in PWS. Recently, a series of 52 patients reported with developmental delay, behavioural changes, epilepsy and congenital heart disease attributable to 15q11.2 microdeletion (BP1-BP2) analysed by array-CGH [10]. Those authors demonstrated augmentation of severity due to size variations of the deletion (type 1).

The case present under study portraysobesity, cognitive impairment, developmental and speech delay as a major phenotype correlating well with DT1. However, the mode of formation of this deletion involving an sSMC and a derivative chromosome resembling a Robertsonian translocation is unusual.

## Conclusion

It is imperative to know the mode of inheritance in caseswhere structural rearrangements in a proband are detected. Additionally, the chances of detecting submicroscopic alterations in child with dysmorphism should not be neglected. This could be attributed to gene/s that has been disrupted at or near the breakpoint region/s using various diagnostic modalities. Also such insights help providing precise genotype-phenotype correlation, management and counselling to patients and families with specific inherited conditions.

## Consent

Written informed consent was obtained from the patient’s parents for publication of this paper and any accompanying images. A copy of the written consent is available for review bythe Editor-in-Chief of this journal.
